# Comparison of Sexual Knowledge between Patients Prepared for Cardiac Surgery and Patients Prepared for Cardiac Rehabilitation in Iran 

**Published:** 2019-07

**Authors:** Behzad Heydarpour, Parvin Ezzati, Ali Soroush, Mozhgan Saeidi, Saeid Komasi

**Affiliations:** 1 *Cardiac Rehabilitation Center, School of Medicine, Imam Ali Hospital, Kermanshah University of Medical Sciences. Kermanshah, Iran.*; 2 *Lifestyle Modification Research Center, Imam Reza Hospital, Kermanshah University of Medical Sciences, Kermanshah, Iran.*; 3 *Clinical Research Development Center, Imam Reza Hospital, Kermanshah University of Medical Sciences, Kermanshah, Iran.*

**Keywords:** *Cardiovascular diseases*, *Cardiac surgical procedures*, *Cardiac rehabilitation*, *Iran*

## Abstract

**Background:** Although sexual death during intercourse occurs rarely in patients with cardiovascular diseases (CVDs), most such patients avoid it because they fear a dangerous event. Given the significance of awareness about this issue among patients with CVDs, we sought to compare sexual knowledge between 2 groups of patients prepared for cardiac surgery and patients prepared for cardiac rehabilitation (CR).

**Methods:** This cross-sectional study, conducted between April and July 2016, recruited 157 patients with CVDs (107 candidates for surgery and 50 patients prepared for CR) in Imam Ali Hospital, in the Iranian city of Kermanshah. The cases, selected through entire counting according to our inclusion criteria, responded to a standard sexual knowledge inventory. Sexual knowledge and professionals responsible in providing sexual rehabilitation from the perspective of patients were compared using the independent t-test and the χ^2 ^test.

**Results:** The participants’ mean age was 55.39±9.82 years (male: 58.6%). Overall, the 2 groups had poor sexual knowledge. Although the CR program started 57.85±13.92 days after surgery, the sexual knowledge of this group of patients was not significantly different from that of the patients prepared for surgery (P=0.904). This difference was not significant between the 2 genders (P=0.077). Finally, concerning the professionals responsible in providing sexual rehabilitation, the patients selected psychologists (P=0.006) and nurses (P=0.012) more frequently in the initial phase of CR program.

**Conclusion:** Sexual knowledge was poor in our CR patients at the outset of the program. Given the poor knowledge in these patients 2 months after surgery and the lack of significant difference in knowledge between these patients and those prepared for surgery, it is advisable that they be provided with the necessary information in this regard in this golden time before hospital discharge.

## Introduction

Cardiovascular diseases (CVDs) are the chief culprits for mortality in men and women the world over in that they account for death in 54% and 43% of European women and men, respectively.^1^ It is predicted that one-fourth of the American population will be afflicted by a type of CVDs by the year 2030.^1^ Patients with CVDs present various complications after the heart event. One of these complications is sexual dysfunction. These patients tend to complain of sexual dysfunction twice as often as do their healthy peers, indicating that sexual activity is liable to decline in coronary patients.^2^ The World Health Organization (WHO) considers sexual dysfunction to be the individual’s inability to participate in a desired sexual relationship and regards it as a significant problem affecting the quality of life.^3^ In Iran, the rate of sexual dysfunction has been reported to be 20.1% and 76.4% before and 12 weeks after surgery, respectively.^4^ Nonetheless, these patients are usually confused about the effects of sexual activity on the heart after the cardiac event.^5^ In other words, although death due to intercourse occurs rarely in patients with CVDs, most patients avoid sexual activity because of fear about sudden cardiac death or re-infarction, dyspnea, anxiety, chest angina, fatigue, change in libido, depression, decreased sexual pleasure, impotency, partner’s anxiety or worry, and feeling of guilt.^6^ In this regard, the results of a study by Schumann et al.^7^ indicated that 23.1% of their patients had no sexual activity before a rehabilitation program.

With respect to sexual activity among patients with CVDs, the caregivers of such patients play an important role in their assessment and the provision of appropriate information and support for the resumption of their sexual activity.^8^ A previous investigation in Iran showed that cardiac health-team members, especially nurses, lacked sufficient knowledge as regards sexual activity in patients with CVDs.^5^ What exacerbates the situation is the personal nature of sexual dysfunction, which compels patients and even physicians to eschew broaching this issue.^9^ Culture and traditional beliefs also impact the individual’s decision to seek treatment for sexual dysfunction. The available statistics show that the rate of seeking treatment for sexual dysfunction is 19% in Brazil and approximately zero in some Asian countries such as Japan.^10^ In Iran, previous studies have paid precious little attention to the evaluation of sexual knowledge and the rate of seeking treatment for such problems on the part of patients with CVDs. Nevertheless, there is some information indicating that a significant portion of the general population with sexual dysfunction refuses to seek and receive professional treatment for their sexual problems.^11^^, ^^12^

Thus, it appears that an assessment of sexual knowledge among patients candidated for cardiac surgery may boost the outcome of future training programs. Indeed, after cardiac surgery, patients’ knowledge about their level of sexual function is of great significance.^13^ However, Lunelli et al.^14^ reported that return to sexual activity after CVDs was an issue generally ignored by caregivers during patients’ hospitalization period. 

According to the abovementioned challenges, we designed the present study to determine the level of sexual knowledge and compare it between patients prepared for cardiac surgery and patients prepared for cardiac rehabilitation (CR), and to compare the 2 groups according to their selection of sexual rehabilitation specialists for the provision of sexual training.

## Methods

The present cross-sectional study, performed from April to July 2016, recruited all patients candidated for cardiac surgery or prepared for a CR program (approximately 2 months after discharge from the hospital for heart surgery) in Imam Ali Hospital, in the Iranian city of Kermanshah. The study population was comprised of 157 patients (107 patients candidated for surgery vs. 50 patients prepared for CR), selected via entire counting. Although there were initially 128 cases candidated for cardiac surgery, only 107 patients fulfilled the study’s inclusion criteria. Moreover, the number of patients in the CR group was lower than that in the surgery group because fewer patients participated in the CR program. A previous study on patients with CVDs in Iran reported that fewer than 15% of the patients entered a CR program.^15^ In our study period, the CR program was commenced for 55 patients, only 50 of whom met our inclusion criteria. In 3 related studies,^16^^-^^18^ an average of 131 patients with CVDs were examined. Therefore, the sample size of the current study appears to be appropriate. The inclusion criteria were comprised of age between 18 and 80 years, fluency in the Persian language, and willingness for participation. Informed consent was obtained from all the participants before study commencement, and the subjects were given reassurances as to the confidentiality of their information. The project received a code of ethics from Kermanshah University of Medical Sciences (ID: KUMS.REC.1394.32).

After primary screening and sample selection based on the inclusion criteria, the research team recorded the demographic characteristics and medical histories of the patients in designed forms after a short interview. The information collected was thereafter matched with the patients’ medical records. If there was a discrepancy between the 2 records, the accuracy of the information was checked by the patient’s cardiologist. Next, the team’s psychologist provided necessary explanations and a sexual knowledge inventory to the patients. The inventories were completed individually by the participants and in the presence of the researcher. The data-collecting instrument was a 10-item sexual knowledge inventory designed by Djurović et al.^13^ (2010). This inventory contains Yes/No questions, and the first 9 questions relate to the time of the resumption of sexual activity, the position of the body during intercourse, appropriate daytimes for intercourse, and symptoms related to cardiac health. Each of these 9 items carries a score of 1, the total score being 9; a higher score, therefore, denotes more sexual knowledge. The last item in the inventory inquires the patient’s viewpoint on the delivery of sexual training by sexual rehabilitation specialists by asking the patient to select 2 health-care professionals with more competence in sexual training. Item 10 does not carry a score. Djurović et al. reported the validity of this inventory to be appropriate.^13^ In the present study, the internal consistency (Cronbach’s alpha) of this questionnaire was 0.65.

At baseline, the data on gender, marital status, education levels, diagnoses and medical processes, medical histories, histories of sexual problems, and medications before the development of CVDs were analyzed using the χ^2 ^test, while the* t*-test was applied to compare age between the 2 groups. In the main analysis, sexual knowledge was compared between the study groups via the *t*-test. Before the *t*-test was run, the non-violation of the statistical assumptions such as data normality was approved. All the analyses were conducted with the SPSS software, version 20, and a P value of less than 0.05 was considered statistically significant. 

## Results

Table 1 depicts the demographic characteristics and the medical histories of the patients in the 2 groups of cardiac surgery and CR program. There were significant differences between the groups in terms of gender (P=0.005), education level (P=0.008), and history of diabetes (P=0.039) at baseline. In addition, according to the results, the mean waiting time before CR commencement was 57.85±13.92 days.

**Figure 1:**
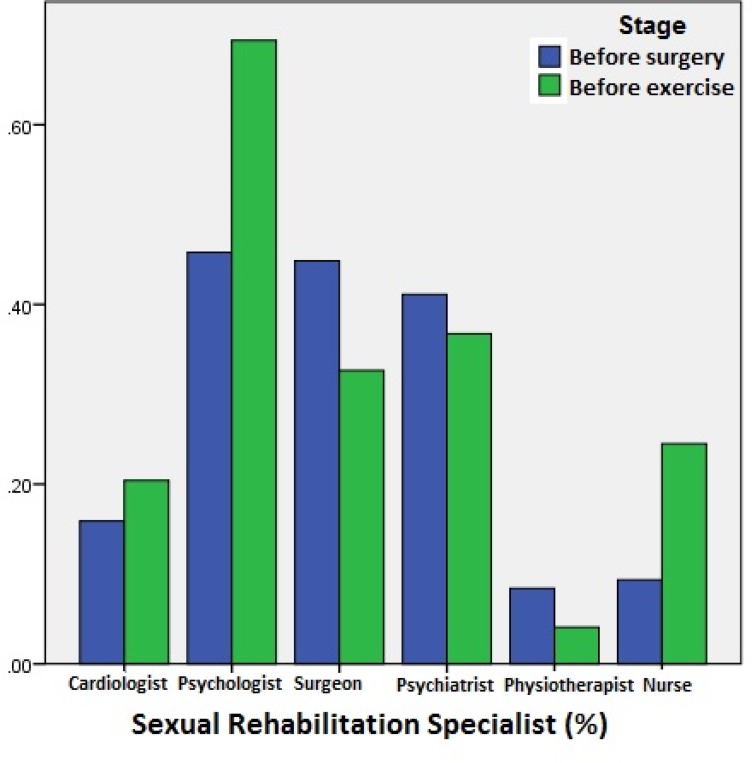
Attitude of the patients concerning sexual rehabilitation specialists responsible for providing sexual rehabilitation services

Table 2 demonstrates the difference in sexual knowledge between the 2 groups of cardiac surgery and CR program. There were no significant differences between the 2 groups (P=0.904). Additionally, there were no significant differences between the genders (P=0.077). Apropos the role of sexual rehabilitation specialists in the provision of sexual knowledge, the patients selected psychologists (P=0.006) and nurses (P=0.012) more frequently in the initial phase of CR program (Figure 1).

**Table 1:** Characteristics of the participants*

	Total (N=157)	Patients Prepared for Surgery (n=107)	Patients Prepared for CR (n=50)	P value ^a^
Age (y)	55.399.82	55.579.44	55.0010.69	0.738
Sex				0.005
Female	65 (41.4)	55 (51.4)	10 (20.0)	
Male	92 (58.6)	52 (48.6)	40 (80.0)	
Marital status				0.563
Married	140 (89.2)	95 (88.8)	45 (90.0)	
Single/Widowed/Separated	17 (10.8)	12 (11.2)	5 (10.0)	
Education Level				0.008
Under diploma	114 (72.6)	86 (80.4)	28 (56.0)	
Diploma	31 (19.7)	16 (14.9)	15 (30.0)	
University qualifications	12 (7.6)	5 (4.7)	7 (14.0)	
Diagnosis				0.380
CABG	135 (86.0)	91 (85.1)	44 (88.0)	
VHS	11 (7.0)	7 (6.5)	4 (8.0)	
Other	11 (7.0)	9 (8.4)	2 (4.0)	
Hypertension	52 (33.1)	41 (38.3)	11 (22.0)	0.051
Diabetes mellitus	46 (29.3)	37 (34.6)	9 (18.0)	0.039
Hyperlipidemia	30 (19.1)	22 (20.6)	8 (16.0)	0.533
Sexual problems before surgery	43 (27.4)	29 (27.1)	14 (28.0)	0.849
Taking sex medication	11 (7.0)	7 (6.5)	4 (8.0)	0.714

CR, Cardiac rehabilitation; CABG, Coronary artery bypass graft; VHS, Valvular heart surgery

* Data are presented as mean±SD or n (%).

**Table 2:** Comparison of the sex knowledge score between the 2 patient groups

	Total (N=157; male=92)	Patients Prepared for Surgery (n=107; male=52)	Patients Prepared for CR (n=50; male=40)	P value
Sexual knowledge	3.691.32	3.701.38	3.671.18	0.904
Female	3.471.31	3.441.37	3.670.87	0.628
Male	3.851.31	3.981.35	3.681.25	0.269
Professionals responsible in providing sexual rehabilitation (%)				
Cardiologist	27 (17.2)	17 (15.9)	10 (20.0)	0.488
Psychologist	83 (52.9)	49 (45.8)	34 (68.0)	0.006
Surgeon	64 (40.8)	48 (44.9)	16 (32.0)	0.150
Psychiatrist	62 (39.5)	44 (41.1)	18 (36.0)	0.603
Physiotherapist	11 (7.0)	9 (8.4)	2 (4.0)	0.327
Nurse	22 (14.0)	10 (9.3)	12 (24.0)	0.012

*Data are presented as mean±SD or n (%).

## Discussion

We performed the present study to compare sexual knowledge between patients prepared for cardiac surgery and patients prepared for CR. The prevalence and severity of sexual problems in patients with CVDs after surgery increase dramatically.^4^ This problem is likely to result from a decrease in sex hormones and chronic illnesses such as hypertension and diabetes.^19^^, ^^20^ Approximately one-third of our patients suffered from hypertension and diabetes.

We found no significant difference in sexual knowledge between our 2 groups. Simply put, sexual knowledge was not enhanced in the patients prepared for CR 2 months after surgery. The absence of difference in sexual knowledge between the 2 groups can be explained in several ways, but what needs to be highlighted is, first and foremost, patients’ role and then health teams’ role. In Iran, sexual dysfunction affects about two-thirds of cardiac surgery patients^4^ and it has been suggested that 23.1% of patients avoid any sexual activity before the CR program.^7^ This is mainly due to the feeling of shame and embarrassment. In other words, patients are liable to feel shame about expressing their worries and sex-related queries in the face of sexual problems. Previous studies have confirmed this hypothesis.^21^^, ^^22^

On the other hand, patients are confronted by a serious challenge as to the selection of professional sources of sexual information delivery. As much as patients welcome the discussion about their sexual problems if physicians broach the subject,^23^ there are very few physicians and nurses who do ask about their patients’ sexual problems^24^ and patients find it difficult to initiate a conversation with health-care professionals about their sexual problems, which stems from cultural and social considerations. The findings of a study in Iran indicated that although approximately three-quarters of cardiologists felt responsible to deliver sexual training to their patients with CVDs, only one-third of them trusted their own knowledge regarding this issue and only 10% of them assessed their patients’ sexual problems frequently.^17^ By no means are nurses an exception to this rule. A previous study showed that almost 87% of nurses agreed that sexual consultation with patients was a part of their duty, but they very rarely conducted it. In addition, 40% of the nurses assessed avoided discussing sexual issues because of fear of inability to answer the patients’ questions.^25^ Whereas between 39% and 52% of nurses in European countries provided patients suffering from CVDs with information about sexual issues,^26^^, ^^27^ only 11% of patients in the Middle East reported that they had received any information or consultation concerning sexual life following cardiac-related problems.^28^ According to previous research, nurses blame a poor sexual knowledge and a feeling of discomfort when discussing sexual issues for the dearth of sexual consultation.^27^^, ^^29^ Previous findings also indicate that nurses have insufficient knowledge about the sexual activity of patients with CVDs^27^ and at least one-fifth of nurses admit the problem of a poor sexual knowledge.^26^ It can, therefore, be concluded that a paucity of sexual knowledge among nurses is one of the serious barriers to dealing with the sexual worries of patients with CVDs.^27^

Nurses’ poor sexual knowledge takes on even more significance in light of the results of the present study regarding the patients’ viewpoints on the health-care professionals responsible for sexual consultation. One of our findings was that the patients prepared for CR, in comparison with those prepared for cardiac surgery, selected psychologists and nurses as the most important sexual rehabilitation specialists for the provision of sexual training. Consequently, psychology and especially, nursing teams should attach greater significance to training for sexual rehabilitation. Indubitably, sexual activity is a normal health function with a significant role in physical health, similar to walking or other daily activities; and it is necessary that specialists pay sufficient heed to this issue and encourage patients to lead an active physical life, including sexual activity.^30^

If patients do not receive appropriate information in the appropriate time, they usually act according to their limited information, fears, beliefs, or superstitions about their sexual relationships.^13^ Indeed, previous research underscores the importance of timely training for patients during their hospital stay.^9^ Moreover, the resumption of sexual activity is deemed an integral component of psychological improvement in patients^4^ and most patients with CVDs demand information about their sexual life.^22^

There a few limitations to the current study. Our study population was recruited from the patients of one single hospital in the west of Iran; a similar study on a larger sample from across the country can confer more robust conclusions. Another salient weakness is that we evaluated 2 groups of patients in 2 different phases of treatment; consequently, factors such as the patients’ past experiences may have led to bias in the findings. We suggest that in future studies, a single group of patients be evaluated at 2 time periods (before surgery and before exercise in CR). Meanwhile, the use of tools with Iranian standardization may yield more reliable information.

## Conclusion

Sexual knowledge among our patients at the outset of the CR program was poor. Given this inadequate knowledge 2 months after surgery and also the lack of a significant difference in knowledge between this group of patients and the group of patients prepared for surgery, it is advisable that these patients be provided with the necessary information in this regard in this golden time before hospital discharge. Psychologists and nurses can play an important role in furnishing information and sexual consultation.

## Notes:

***This paper should be cited as: ***Heydarpour B, Ezzati P, Soroush A, Saeidi M, Komasi S. Comparison of Sexual Knowledge between Patients Prepared for Cardiac Surgery and Patients Prepared for Cardiac Rehabilitation in Iran. J Teh Univ Heart Ctr 2019;14(3):103-108.
